# FGF2 Attenuates Neural Cell Death via Suppressing Autophagy after Rat Mild Traumatic Brain Injury

**DOI:** 10.1155/2017/2923182

**Published:** 2017-10-17

**Authors:** Chonghui Tang, Yudong Shan, Yilan Hu, Zhanjian Fang, Yun Tong, Mengdan Chen, Xiaojie Wei, Xiaojun Fu, Xinlong Xu

**Affiliations:** Department of Neurosurgery, Affiliated Cixi Hospital, Wenzhou Medical University, No. 999 Nanerhuandong Road, Ningbo 315300, China

## Abstract

Traumatic brain injury (TBI) can lead to physical and cognitive deficits, which are caused by the secondary injury process. Effective pharmacotherapies for TBI patients are still lacking. Fibroblast growth factor-2 (FGF2) is an important neurotrophic factor that can stimulate neurogenesis and angiogenesis and has been shown to have neuroprotective effects after brain insults. Previous studies indicated that FGF2's neuroprotective effects might be related to its function of regulating autophagy. The present study investigated FGF2's beneficial effects in the early stage of rat mild TBI and the underlying mechanisms. One hundred and forty-four rats were used for creating controlled cortical impact (CCI) models to simulate the pathological damage after TBI. Our results indicated that pretreatment of FGF2 played a neuroprotective role in the early stage of rat mild TBI through alleviating brain edema, reducing neurological deficits, preventing tissue loss, and increasing the number of surviving neurons in injured cortex and the ipsilateral hippocampus. FGF2 could also protect cells from various forms of death such as apoptosis or necrosis through inhibition of autophagy. Finally, autophagy activator rapamycin could abolish the protective effects of FGF2. This study extended our understanding of FGF2's neuroprotective effects and shed lights on the pharmacological therapy after TBI.

## 1. Introduction

Traumatic brain injury (TBI), the leading cause of death and disability nowadays, is a major health problem all over the world [1, 2]. Even mild TBI can cause delayed physical and cognitive deficits [3]. Although lots of randomized controlled trials (RCTs) were done in recent years, no intervention had shown to be beneficial [4–6]. Thus, it is imperative to further elucidate the complicating pathophysiological mechanisms of TBI and develop effective pharmacological intervention targets. It is generally acknowledged that TBI has two injury phases—primary injury and secondary injury. The primary injury is directly caused by trauma itself, while the secondary injury is more complex with a series of pathological responses, including blood-brain barrier disruption, oxidative stress, neuroinflammation, autophagy, apoptosis, and necrotic cell death. These processes in the secondary injury are directly related with long-term neurological deficits and also provide us multiple therapeutic targets in TBI management.

Autophagy is a lysosomal degradation pathway that protects organisms against diverse pathologies [7]. In most circumstances, autophagy could promote cell survival by maintaining cellular homeostasis, but there are a number of studies that demonstrated that autophagy could also trigger cell death in certain pathological situations [8–10]. Researchers had already observed autophagy's existence in TBI model several years ago, and modulation of this process could result in neurological improvements [11–13]. However, up until recent years, whether inducing or inhibiting autophagy can result in neuroprotection remains controversial [14–16]. The relationship between autophagy and other forms of cell death such as apoptosis after TBI is worth studying.

Fibroblastic growth factors (FGFs) are small polypeptide growth factors which play a pivotal role in morphogenesis [17]. Fibroblast growth factor-2 (FGF2), also known as basic fibroblast growth factor (bFGF), is an important member of this family. FGF2 is highly expressed in the central nervous system and exhibits early decline during the course of aging [18]. Previous studies showed that it had a variety of neuroprotective effects, including supporting neural stem and progenitor cell proliferation in vitro and in vivo [19, 20], maintaining vascular integrity and angiogenesis [21, 22], and helping cognitive recovery [20]. Studies have demonstrated the neuroprotective roles of FGF2 in various pathological conditions in the central nervous system, such as TBI, spinal cord injury (SCI), ischemic brain injury, subarachnoid hemorrhage (SAH), and neurodegenerative diseases [21, 23–26]. Most of the previous studies focused on neurogenesis effects of FGF2, but several recent studies have discovered FGF2's novel effect as an autophagy inhibitor, in which the activation of PI3K/Akt/mTOR signaling pathway may take an important part [27, 28]. As the molecular mechanisms of FGF2 in treatment of TBI has not been fully understood, we hypothesized that FGF2 could also inhibit autophagy and attenuate cell death in TBI treatment.

In the present study, we found that FGF2 could act as a neuroprotective agent after rat mild TBI, alleviating brain edema, reducing cerebral lesion volume, and promoting functional recovery. Meanwhile, FGF2 also inhibited autophagy and decreased neural apoptosis and necrotic cell death. Furthermore, autophagy activator rapamycin could abolish the protective effects of FGF2. These results provide us a new perspective about FGF2's neuroprotective role after TBI.

## 2. Materials and Methods

### 2.1. Animals and Study Design

Adult male Sprague-Dawley rats (250–300 g) obtained from SLAC Laboratory Animal Co. Ltd. (Shanghai, China) were used in this study. The animals were maintained under controlled temperature and humidity conditions at a 12 hr light/dark cycle place. All procedures involving animals were strictly conformed to the *Guide for the Care and Use of Laboratory Animals* of the National Institutes of Health and approved by the Institutional Animal Care and Use Committee of Wenzhou Medical University.

### 2.2. CCI Model

The procedure details used for creating CCI model were described previously [29]. Briefly, after the rats were anesthetized with pentobarbital (40 mg/kg) through intraperitoneal injection, the head of them was fixed on a stereotaxic frame. A 5 × 5 mm craniotomy was performed approximately midway between the bregma and lambda on the right frontoparietal cortex after a midline incision. The bone flap was then removed carefully without disturbing the underlying dura. Then the CCI was performed using a PinPoint™ Precision Cortical Impactor (Cary, NC, USA) perpendicular to the brain surface (12° from the vertical). The impact tip was 4 mm in diameter, with an impact velocity of 3 m/s, a duration time of 120 ms, and a deformation depth of 2 mm below the dura, to mimic a mild focal TBI in rats. The bone flap was put back and sealed immediately, and the wound was sutured. Rats in the sham group received the same surgical procedure only without CCI. During the surgery, body temperature was monitored and maintained at 37.0 ± 0.5°C with a rectal probe coupled to a heating pad. For arterial pH, pO_2_, pCO_2_, and blood glucose level monitoring catheterize the right femoral artery before the procedure. The rats were returned to their home cages after completely recovered from anesthesia in a heated chamber.

### 2.3. Experimental Design

#### 2.3.1. Experiment 1

To explore the effects of FGF2 in rat TBI models, ninety rats were randomly assigned into three groups: the sham group (*n* = 30), TBI + vehicle group (*n* = 30), and TBI + FGF2 group (*n* = 30). A dose of 250 *μ*g/kg recombinant human FGF2 (PeproTech Inc., Rocky Hill, NJ, USA) was administrated intranasally 1 h before TBI induction in the TBI + FGF2 group. Intranasal administration could deliver FGF2 directly to the brain through nasal epithelium [30, 31]. The dosage and time point of FGF2 pretreatment were based on a previous study [22]. The sham group and TBI + vehicle group received the same volume of sterile saline intranasally at the same time point before TBI induction. The endpoints of the experiments were 6 h (PI staining) and 48 h (other experiments) after TBI induction, based on a previous study [32]. Six rats in each group were used for brain water content assessment, lesion volume assessment, Western blot, immunofluorescence staining, and PI staining.

#### 2.3.2. Experiment 2

To explore whether autophagy mediates FGF2 effects, fifty-four rats were randomly assigned into three groups: the TBI + vehicle group (*n* = 18), TBI + FGF2 group (*n* = 18), and TBI + FGF2 + Rap group (*n* = 18). The dosage and time point of FGF2 pretreatment were the same as in experiment 1. A dosage of 2 mg/kg rapamycin (Selleck Chemicals, Houston, TX, dissolved in 2% DMSO) was administrated intraperitoneally 30 min after TBI induction in the TBI + FGF2 + Rap group based on a previous study [33]. The TBI + vehicle group received same volume of sterile saline intranasally 1 h before TBI induction. The TBI + vehicle group and TBI + FGF2 group received the same volume of 2% DMSO intraperitoneally 30 min after TBI induction. Six rats in each group were used for Western blot, immunofluorescence staining, and PI staining. The endpoints of the experiments were the same as in experiment 1.

### 2.4. Evaluation of Neurological Deficits

To examine the effects of FGF2 on the neurological deficits of animals after TBI, the modified neurological severity scores (mNSS) were used. The neurological scores of the animals in each group were evaluated by an independent observer at 48 h after TBI induction using motor, sensory, reflex, and balance tests. A total score ranging from 0 to 18 was calculated by adding the scores together, and a higher score indicated a worse neurological function [34]. Most mNSS of the rats were lower than 6, which indicated a mild injury after TBI. Foot fault test was examined in experiment 2 to evaluate the cognitive functional deficits by an independent observer at 48 h after TBI induction. The rats were put on an elevated grid and allowed to move freely for 5 minutes or until 50 steps were taken with the left forelimb. The total number of steps and fell time of the left forelimb were counted, and the percentage of left forelimb foot faults was calculated.

### 2.5. Brain Water Content Measurement

Rats were sacrificed under deep anesthesia after 48 h of TBI. The brains were removed, and the right hemispheres were collected immediately. After weighing for the wet weight, the samples were then dried at 105°C for 24 h to obtain the dry weight. The brain water content was calculated using the following formula: (wet weight − dry weight)/wet weight × 100%.

### 2.6. Hematoxylin and Eosin (HE) Staining and Lesion Volume Assessment

At 48 h after TBI, the rats were sacrificed and perfused intracardially with 0.1 mmol PBS (pH, 7.4) and 4% paraformaldehyde (pH, 7.4). The brains were then removed and immersed in the same perfusate at 4°C for 72 h. Coronal sections started from one millimeter which was anterior to the lesion margin, to the posterior lesion margin, were obtained and then paraffin embedded. The lesion volumes were calculated by integrating 10 sections (3 *μ*m thick) with 300 *μ*m intervals. After HE-staining with standard methods (0.5% hematoxylin, 25°C, 5 min and 0.5% eosin, 25°C, 1 min), the brain sections were imaged using a stereomicroscope (Olympus BX51, Olympus, Tokyo, Japan) in a bright-field illumination and measured using Image-Pro Plus 6.0 (Media Cybernetics, Silver Spring, MD, USA). Lesion volume was calculated as follows: Σ(*A*_*n*_ + *A*_*n*+1_) × *d*/2, where *A* is the ventricular area and *d* is the distance between sections, according to a previously published work [35].

### 2.7. Western Blot

Western blotting was performed as previously described [36]. Briefly, the cortical regions of the brains were collected and homogenized. The samples were centrifuged at 1000*g* for 10 min at 4°C. The supernatant was further centrifuged, and the protein content was measured using a detergent-compatible protein assay kit (Bio-Rad, Hercules, CA, USA). Equal quantities of protein (40 *μ*g) from each samples were resuspended in loading buffer, denatured at 95°C for 5 min, and loaded into the walls of the sodium dodecyl sulfate polyacrylamide gel electrophoresis. After electrophoresis, the protein was transferred onto polyvinylidene fluoride membranes. The membranes were blocked with nonfat dry milk buffer for 2 h and subsequently incubated overnight at 4°C with the following primary antibodies: LC3 (1 : 1000, Cell Signaling Technology CST#4108), Beclin-1 (1 : 1000, Abcam ab62557), p62 (1 : 1000, Abcam ab56416), and *β*-actin (1 : 2000, Santa Cruz SC-47778). The membranes were processed using the appropriate secondary antibodies (1 : 5000) for 1 h at room temperature. The protein band densities were detected using X-ray film, and the densitometric signals were quantified using ImageJ software (NIH, Bethesda, MD, USA).

### 2.8. TUNEL and Immunofluorescence Staining

The rats were sacrificed at 48 h after TBI induction in deep anesthesia and perfused intracardially with 0.1 mmol PBS (pH, 7.4) and 4% paraformaldehyde (pH, 7.4). The brains were then removed and immersed in the same perfusate at 4°C for 72 h and then dehydrated with 30% sucrose solution until they sank to the bottom (about 2 days). Coronal sections (7 *μ*m) were collected from the right hemisphere. The brain sections were incubated with 10% normal goat serum and 0.3% Triton X-100 to prevent nonspecific binding for 1 h at room temperature. Then the sections were incubated at 4°C overnight with the primary antibodies: NeuN (1 : 250, Millipore MAB377) or LC3 (1 : 200, Cell Signaling Technology CST#4108). After washing with 0.01 mmol PBS several times, the sections were incubated with the secondary antibodies for 2 h at 4°C in the dark. A TUNEL staining kit (In Situ Cell Death Detection Kit, Fluorescein, Roche, Switzerland) was used to analyze apoptotic cell death. The brain sections were incubated with TUNEL reaction mixture (90 min) at 37°C. After washing with PBS again, the sections were mounted onto slides with Fluoroshield™ with DAPI (Sigma-Aldrich, St. Louis, MO, USA). Immunostaining was observed using a fluorescent microscope (Olympus, Tokyo, Japan). The total number of NeuN-positive, LC3-positive, or TUNEL-positive cells were counted in three different slices per animal around the lesion area or in the CA1 and CA3 regions of the ipsilateral hippocampus by an independent observer.

### 2.9. Propidium Iodide (PI) Labeling

At 5 h after TBI induction, PI (10 mg/mL; Sigma-Aldrich, St. Louis, MO, USA) was diluted in normal saline and injected to rats intraperitoneally at a dose of 30 mg/kg. After 1 h, the rats were sacrificed and perfused intracardially with 0.1 mmol PBS (pH, 7.4) and 4% paraformaldehyde (pH, 7.4). Then the brains were removed and immersed in 4% paraformaldehyde for 72 h. After dehydrated with 30% sucrose solution, coronal sections (7 *μ*m) were collected from the right hemisphere and PI-positive cells were quantitated around the lesion area or in the CA1 region from 200x cortical fields in three brain sections per rats. The counting task was also done by an independent observer.

### 2.10. Statistical Analysis

To facilitate comparisons between three groups, the Western blotting results were expressed as relative density of the band as compared *β*-actin and then normalized to the mean value of the sham group. The data were expressed as mean ± SD. SPSS Statistics (version 22.0) was used for statistical analysis. All the data were performed using one-way analysis of variance (ANOVA) followed by Tukey's multiple comparison test. Statistical significance was inferred at *P* < 0.05.

## 3. Results

### 3.1. Physiological Evaluation

All the physiological parameters were monitored during the TBI procedure. The body temperature, arterial pH (7.35–7.45), pO_2_ (85–95 mmHg), pCO_2_ (35–45 mmHg), and blood glucose level (95–125 mg/dL) were monitored and analyzed between different groups, and no significant difference exists among groups (data not shown). No mortality was recorded during the experiment.

### 3.2. FGF2 Alleviates Neurological Deficits and Brain Edema

**Figure 1:**
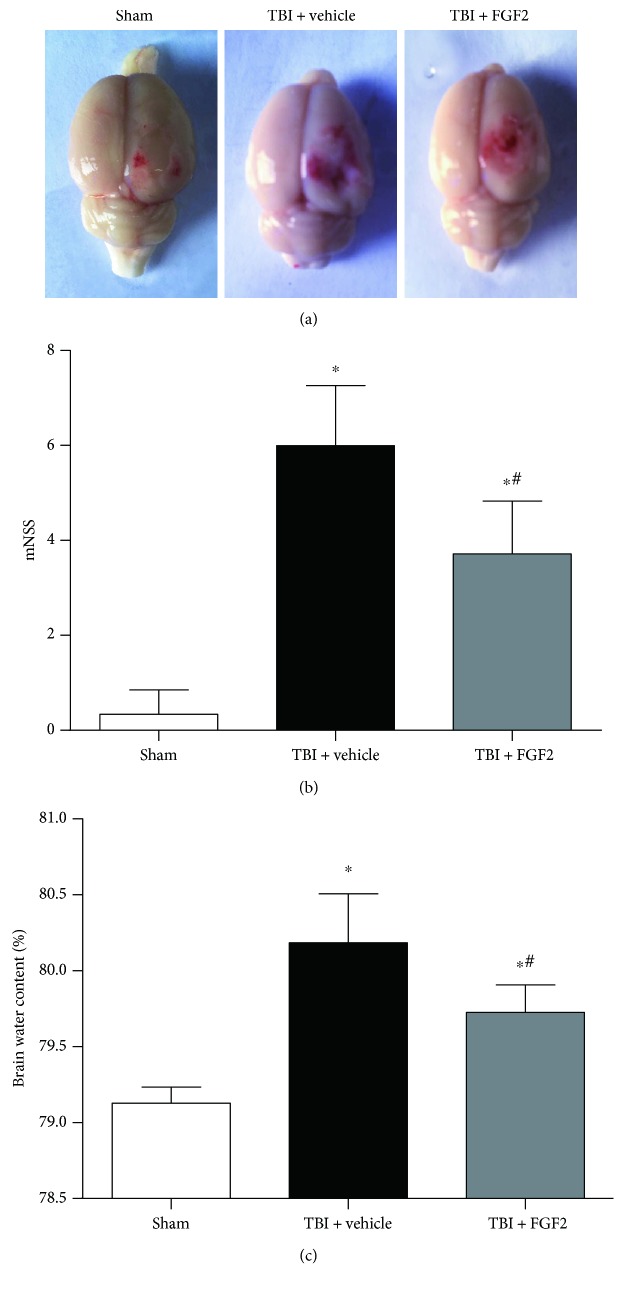
Representative pictures of the brains from each group, mNSS, and brain water content at 48 h after rat mild TBI. (a) Typical brains from the sham, TBI + vehicle, and TBI + FGF2 groups. (b) The quantification of mNSS at 48 h after TBI induction. The *bars* represent the mean ± SD. *n* = 24. ^∗^*P* < 0.05 versus sham and ^#^*P* < 0.05 versus TBI + vehicle. (c) The quantification of brain water content of the right hemisphere. The *bars* represent the mean ± SD. *n* = 6. ^∗^*P* < 0.05 versus sham, ^#^*P* < 0.05 versus TBI + vehicle.

The representative pictures of the brain samples in each group are presented in Figure 1(a). Significant neurological function deficits were observed 48 h after TBI induction (*P* < 0.05, Figure 1(b)). The mNSS were significantly lower in the TBI + FGF2 group than in the TBI + vehicle group (*P* < 0.05, Figure 1(b)). The brain water content of the injured hemisphere was also significantly increased at 48 h post-TBI, pretreatment with FGF2 could attenuate this increase (*P* < 0.05, Figure 1(c)).

### 3.3. FGF2 Prevents the Loss of the Brain Tissue and Promotes Neuronal Survival after TBI

**Figure 2:**
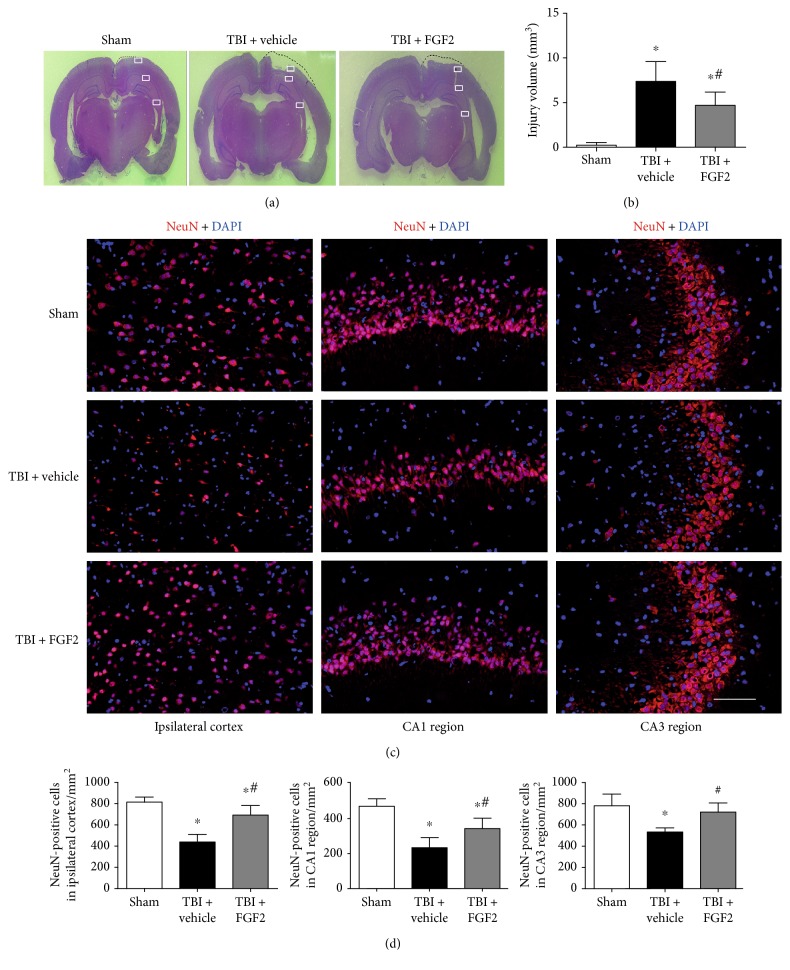
FGF2 prevents the loss of the brain tissue and promotes neuronal survival after TBI. (a) Representative images of HE-stained coronal sections 48 h post-TBI. The white squares indicated the region of the images presented in Figure 2(c). (b) Quantitative analysis of cerebral lesion volume revealed a significant decrease in loss of tissue following treatment with FGF2. The *bars* represent the mean ± SD. *n* = 6. ^∗^*P* < 0.05 versus sham and ^#^*P* < 0.05 versus TBI + vehicle. (c) Representative images of the ipsilateral cortex (left) and CA1 and CA3 regions of the ipsilateral hippocampus (middle and right) on NeuN-stained coronal sections from each group 48 h post-TBI. *Scale bar* = 100 *μ*m. (d) Quantitative analysis revealed significant increases in the numbers of NeuN-positive cells in the ipsilateral cortex (left) and CA1 and CA3 regions of the ipsilaterial hippocampus (middle and right) 48 h post-TBI following pretreatment with FGF2. The *bars* represent the mean ± SD. *n* = 6. ^∗^*P* < 0.05 versus sham and ^#^*P* < 0.05 versus TBI + FGF2.

The cerebral lesion volume and the neuronal survival were assessed at 48 h post-TBI. The representative coronal sections in each group are shown in Figure 2(a). The lesion volumes of the TBI + FGF2 group were significantly smaller than those of the TBI + vehicle group at 48 h (*P* < 0.05, Figure 2(b)). The numbers of NeuN-positive cells in the ipsilateral cortex and the CA1 and CA3 regions of the ipsilateral hippocampus were significantly decreased 48 h after TBI, and this process could be significantly reversed when pretreated with FGF2 (*P* < 0.05, Figures 2(c) and 2(d)).

### 3.4. FGF2 Prevents the Increase of LC3-Positive Cell Numbers after TBI

**Figure 3:**
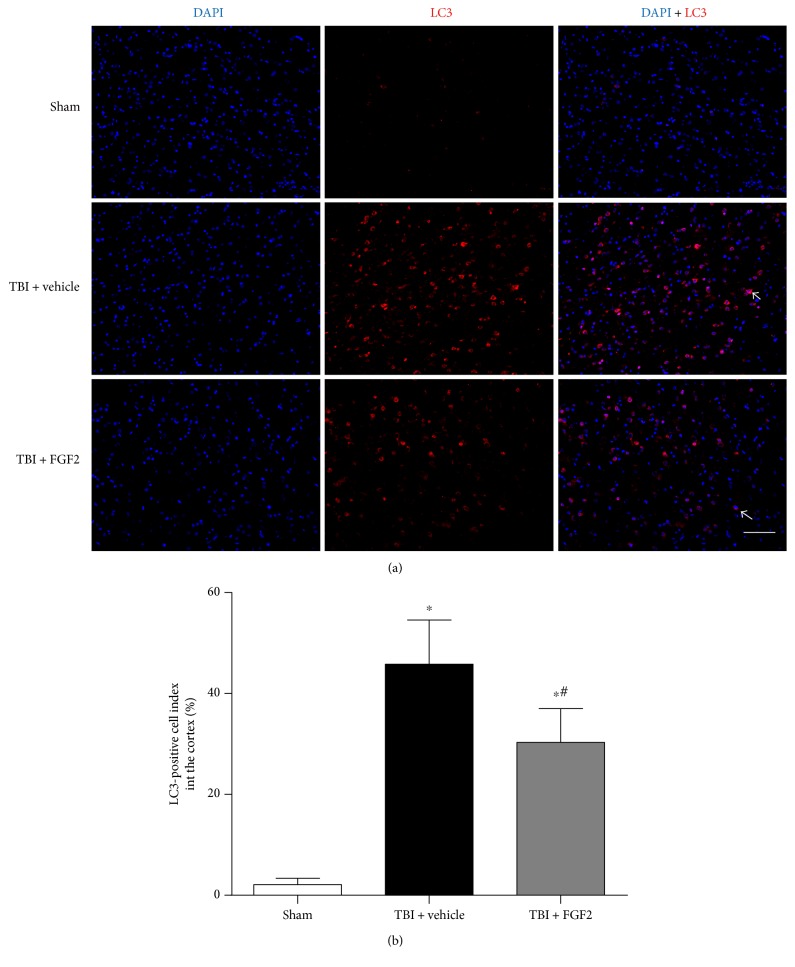
Effects of FGF2 on the expression of LC3 in the ipsilateral cortex 48 h post-TBI. (a) Representative microphotographs showed the colocalization of DAPI (blue) with LC3 (red) positive cells in the ipsilateral cortex 48 h post-TBI. *Scale bar* = 100 *μ*m. (b) The quantification of the LC3-positive cells as a percent of the total DAPI+ cells. The *bars* represent the mean ± SD. *n* = 6. ^∗^*P* < 0.05 versus sham and ^#^*P* < 0.05 versus TBI + FGF2.

Autophagy takes an important part in brain injury after TBI, but the role of FGF2 in regulating autophagy after TBI has not been studied. As LC3 is an ideal biomarker for autophagy activation, we chose LC3 to mark the autophagic cells. The immunofluorescence staining showed that LC3-positive cells could be hardly observed in the cortex of the control group but significantly increased 48 h after TBI induction (*P* < 0.05, Figures 3(a) and 3(b)). In the TBI + FGF2 group, the number of LC3-positive cells was less than that in the TBI + vehicle group, which was statistically significance (*P* < 0.05, Figures 3(a) and 3(b)).

### 3.5. FGF2 Regulates the Autophagy-Related Protein Levels

**Figure 4:**
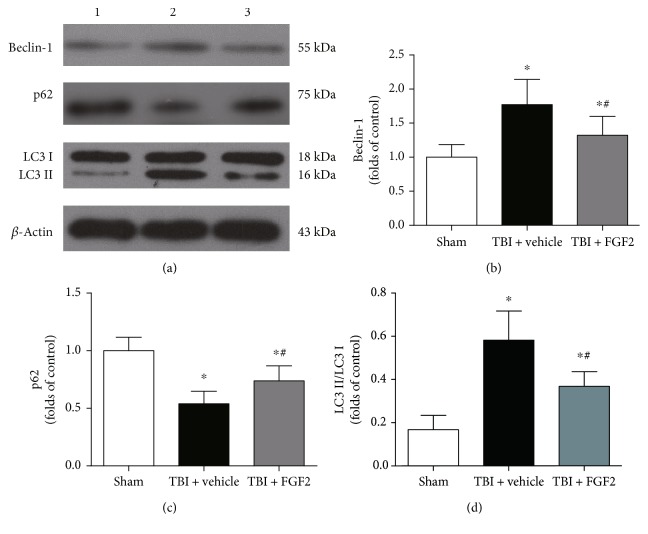
Effects of FGF2 on the expression of autophagy-related proteins in the ipsilateral cortex 48 h post-TBI in different groups. (a) Representative Western blots showing levels of Beclin-1, p62, and LC3 in the ipsilateral cortex 48 h post-TBI. Lane 1, sham group; lane 2, TBI + vehicle group; lane 3 TBI + FGF2 group. (b, c) The relative band densities of Beclin-1 and p62. The densities of the protein bands were analyzed and normalized to *β*-actin. The data are expressed as a percentage of the sham group. The *bars* represent the mean ± SD. *n* = 6. ^∗^*P* < 0.05 versus sham and ^#^*P* < 0.05 versus TBI + vehicle. (d) The band density ratio of LC3 II to LC3 I. The *bars* represent the mean ± SD. *n* = 6. ^∗^*P* < 0.05 versus sham and ^#^*P* < 0.05 versus TBI + vehicle.

To confirm the inhibition effects of FGF2 on autophagy post-TBI, we further measured the protein levels of Beclin-1 and p62 and the ratio of LC3II/I in each group. As shown in Figure 4, the protein levels of Beclin-1 was significantly upregulated in the injured cerebral cortex 48 h after TBI (*P* < 0.05, Figures 4(a) and 4(b)), and FGF2 pretreatmet significantly decreased the Beclin-1 level compared with the TBI + vehicle group (*P* < 0.05, Figures 4(a) and 4(b)). TBI significantly decreased the protein level of p62 at 48 h (*P* < 0.05, Figures 4(a) and 4(c)), while FGF2 could significantly prevent this process (*P* < 0.05, Figures 4(a) and 4(c)). FGF2 pretreatment also significantly upregulated the ratio of LC3II/I compared with the TBI + vehicle group (*P* < 0.05, Figures 4(a) and 4(d)). These results indicated that FGF2 pretreatment could inhibit the level of autophagy at 48 h after TBI.

### 3.6. FGF2 Prevents Cell Apoptosis

**Figure 5:**
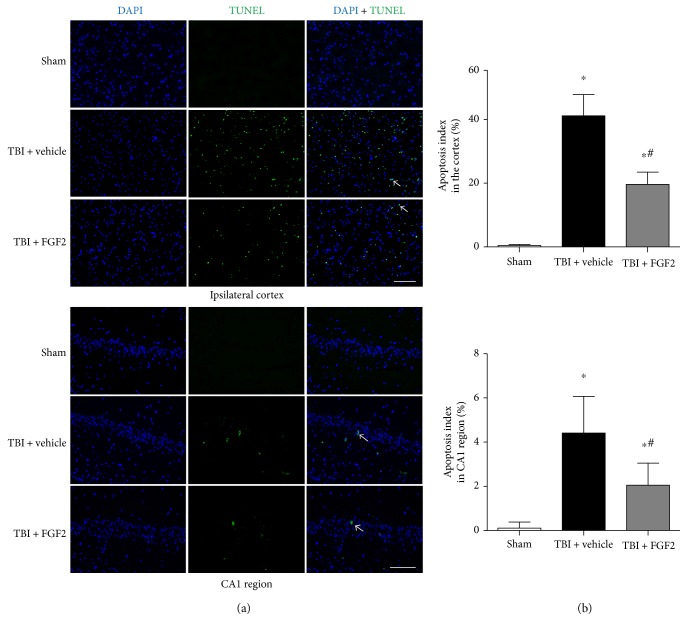
Cortical cellular apoptosis in the ipsilateral cortex 48 h post-TBI in different groups. (a) Representative microphotographs showed the colocalization of DAPI (blue) with TUNEL-positive (green) cells in the ipsilateral cortex and CA1 region 48 h post-TBI. Scale bar = 100 *μ*m. (b) The quantification of the TUNEL-positive cells as the apoptosis index as a percent of the total DAPI+ cells. The bars represent the mean ± SD. *n* = 6. ^∗^*P* < 0.05 versus sham and ^#^*P* < 0.05 versus TBI + vehicle.

TUNEL staining was used to detect the apoptotic cell death in the injured cortex and CA1 region of the ipsilateral hippocampus. In the sham group, TUNEL-positive cells barely exist (Figure 5(a)). TBI significantly increased the apoptotic index compared with the sham group, while pretreatment of FGF2 could significantly prevent the increase of the apoptotic index compared with the TBI + vehicle group (*P* < 0.05, Figures 5(a) and 5(b)).

### 3.7. FGF2 Decreased the Number of Necrotic Cells after TBI

**Figure 6:**
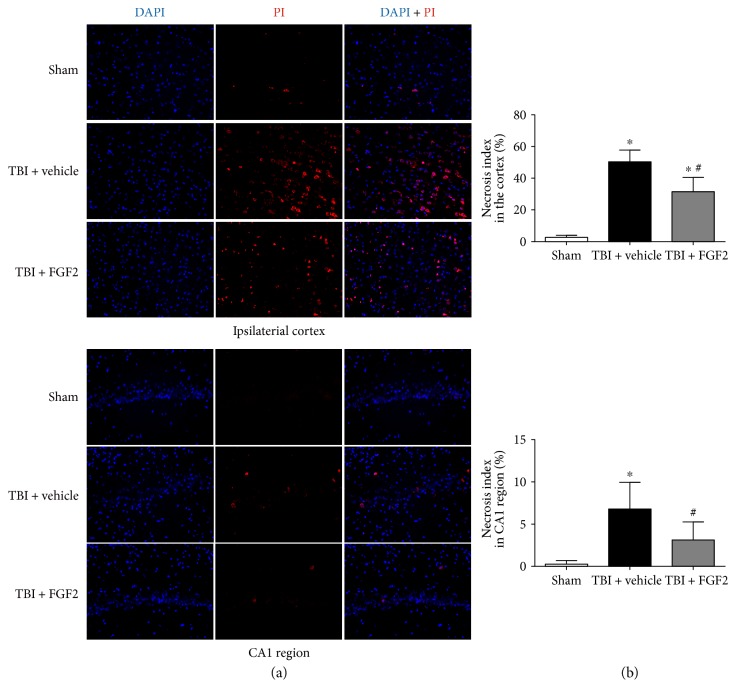
PI-positive cells in the ipsilateral cortex 6 h post-TBI in different groups. (a) Representative microphotographs showed the colocalization of DAPI (blue) with PI-positive (red) cells in the ipsilateral cortex and CA1 region 6 h post-TBI. *Scale bar* = 100 *μ*m. (b) The quantification of the PI-positive cells as the necrosis index as a percent of the total DAPI+ cells. The *bars* represent the mean ± SD. *n* = 6. ^∗^*P* < 0.05 versus sham and ^#^*P* < 0.05 versus TBI + vehicle.

To evaluate the effect of FGF2 in protecting cells from injury, PI was used to identify the plasma membrane disrupted cells. As plasma membrane disruption is one of the key hallmarks of necrotic cell death, PI-positive cells could represent necrotic cell death. The number of PI-positive cells was significantly increased in the injured cortex and CA1 region of the ipsilateral hippocampus in the TBI + vehicle group 6 h after TBI (*P* < 0.05, Figures 6(a) and 6(b)). Pretreatment with FGF2 could significantly prevent the increase of the percentage of PI-positive cells (*P* < 0.05, Figures 6(a) and 6(b)).

### 3.8. Rapamycin Abolished the Neuroprotective Effects of FGF2

**Figure 7:**
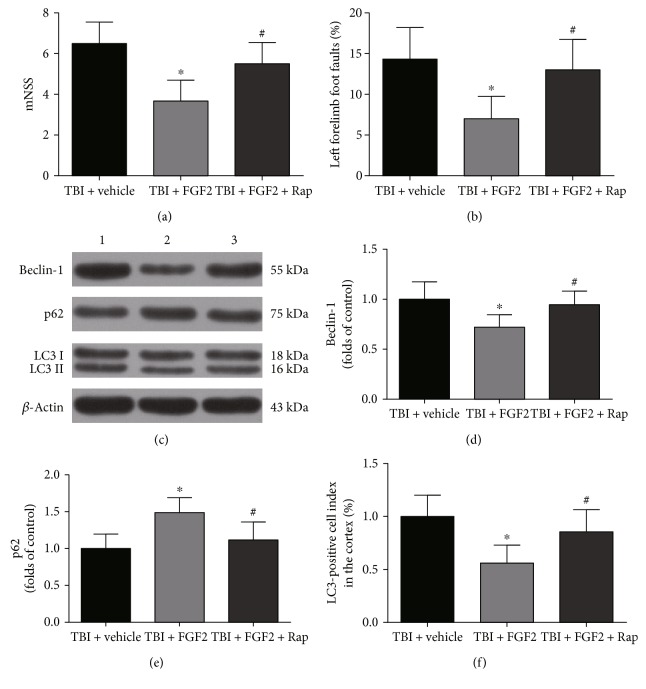
Rapamycin reversed the beneficial effects of FGF2 on neurological function and changed the expression of autophagy-related proteins in the ipsilateral cortex 48 h post-TBI. (a, b) The quantification of mNSS and the percentage of left forelimb foot faults at 48 h after TBI induction. The *bars* represent the mean ± SD. *n* = 24. ^∗^*P* < 0.05 versus TBI + vehicle and ^#^*P* < 0.05 versus TBI + FGF2. (c) Representative Western blots showing levels of Beclin-1, p62, and LC3 in the ipsilateral cortex 48 h post-TBI. Lane 1, TBI + vehicle group; lane 2, TBI + FGF2 group; lane 3 TBI + FGF2 + Rap group. (d, e) The relative band densities of Beclin-1 and p62. The densities of the protein bands were analyzed and normalized to *β*-actin. The data are expressed as a percentage of the sham group. The *bars* represent the mean ± SD. *n* = 6. ^∗^*P* < 0.05 versus TBI + vehicle, ^#^*P* < 0.05 versus TBI + FGF2. (f) The band density ratio of LC3 II to LC3 I. The *bars* represent the mean ± SD. *n* = 6. ^∗^*P* < 0.05 versus TBI + vehicle and ^#^*P* < 0.05 versus TBI + FGF2.

**Figure 8:**
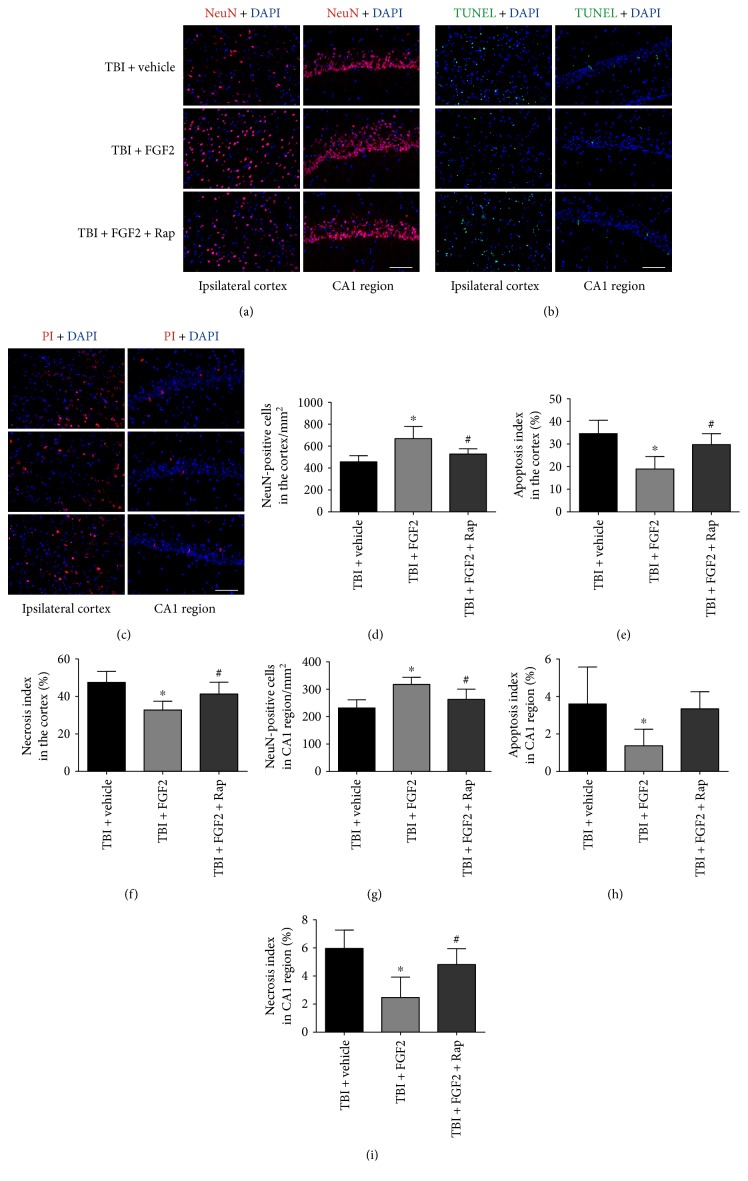
Rapamycin reversed the beneficial effects of FGF2 on neuronal survival and cell death after TBI. (a–c) Representative images of the ipsilateral cortex (left) and CA1 region of the ipsilateral hippocampus (right) on NeuN-stained, TUNEL-stained, and PI-stained coronal sections from each group 48 h post-TBI. *Scale bar* = 100 *μ*m. (d–i) Quantitative analysis of the numbers of NeuN-positive, TUNEL-positive, and PI-positive cells in the ipsilateral cortex and CA1 region of the ipsilaterial hippocampus 48 h post-TBI in each group. The *bars* represent the mean ± SD. *n* = 6. ^∗^*P* < 0.05 versus TBI + vehicle and ^#^*P* < 0.05 versus TBI + FGF2.

To explore whether autophagy mediates FGF2 effects, rapamycin was used to counteract the autophagy inhibition caused by FGF2. The mNSS and foot faults were significantly lower in the TBI + FGF2 group than those in the TBI + vehicle group (*P* < 0.05, Figures 7(a) and 7(b)). The increase of the neurological function with FGF2 management was significantly suppressed by rapamycin (*P* < 0.05, Figures 7(a) and 7(b)). The protein level of Beclin-1 was significantly downregulated in the injured cortex in the TBI + FGF2 group (*P* < 0.05, Figures 7(c) and 7(d)), and rapamycin management significantly increased Beclin-1 level compared with the TBI + FGF2 group (*P* < 0.05, Figures 7(c) and 7(d)). FGF2 significantly increased the protein level of p62 at 48 h (*P* < 0.05, Figures 7(c) and 7(e)), while rapamycin could significantly prevent this process (*P* < 0.05, Figures 7(c) and 7(e)). Rapamycin also significantly upregulated the ratio of LC3II/I compared with the TBI + FGF2 group (*P* < 0.05, Figures 7(c) and 7(f)). Rapamycin could also significantly decrease the NeuN-positive cells in the injured cortex and CA1 region, compared with the TBI + FGF2 group (*P* < 0.05, Figures 8(a), 8(d), and 8(g)). The apoptosis index and necrosis index in the injured cortex were also increased after rapamycin management (*P* < 0.05, Figures 8(b), 8(c), 8(e), and 8(f)). The necrosis index of ipsilateral CA1 region in the TBI + FGF2 + Rap group was also significantly higher than that in the TBI + FGF2 group (*P* < 0.05, Figures 8(c) and 8(i)). These results indicated that rapamycin could reverse the downregulation of the autophagy level caused by FGF2 and abolish the neuroprotective effects of FGF2, demonstrated that reduced autophagy mediates FGF2-induced benefit effects after rat mild TBI.

## 4. Discussion

In the present study, we explored the neuroprotective role of FGF2 in rat mild TBI model and studied the potential mechanisms. We found that pretreatment with FGF2 had neuroprotective effect in rat mild TBI by alleviating neurological deficits, decreasing brain water content and reducing lesion volume. FGF2 also had the ability to increase the number of surviving neurons at the site of injury. Furthermore, we discovered the role of autophagy in FGF2 management after rat mild TBI for the first time. FGF2 could significantly decrease the apoptotic cell death and necrotic cell death through inhibiting autophagy after TBI. As an autophagy activator, rapamycin could reverse the neuroprotective effects of FGF2.

As previously mentioned, the long-term consequence of TBI was dominated by the secondary injury, so the key to successful treatment had to focus on how to alleviate secondary damage after TBI [37]. Brain edema and the increased intracranial pressure in the early stage of TBI are the main causes of mortality, even a minor increase of brain water content can lead to a significant increase of intracranial pressure and the poor outcome [38]. Thus, alleviate brain edema is a promising pharmacological therapeutic direction in TBI management.

The concentration of FGF2 significantly increases in the interstitial space in the first week after TBI [39]. FGF2 binds to the fibroblast growth factor receptor 1 (FGFR1) which is located on the cell membrane of neurons [40] and stimulates the neural stem/progenitor cell proliferation and differentiation [20]. However, the fact is that for TBI patients, spontaneous improvement of neurologic function is limited and almost all of them will keep a stable condition after 12 months. Exogenous FGF2 had been used as a pharmacological intervention method in experimental TBI since many years ago [41]. However, most of the previous studies focused on its delayed effect of stimulating neurogenesis [20, 21], and to our knowledge, there is only one research about FGF2's effects in the early stage of TBI that is focused on its effect of alleviating blood-brain barrier disruption through activation of PI3K/Akt/Rac1 pathway [22]. Although disruption of blood-brain barrier permeability is the main cause of vasogenic edema [37], no direct evidence about FGF2's effect in reducing brain water content after TBI could be found in the literature. So, in this study, we tested FGF2's neuroprotective effects in the first 48 h after TBI and found that it could significantly alleviate brain water content of the injured hemisphere and promote mNSS of the injured rats. These results might be due to the alleviation of blood brain barrier disruption and were consistent with previous studies about FGF2's neuroprotective effects after intracerebral hemorrhage and ischemic brain injury [24, 42]. We also found that FGF2 could prevent tissue loss and increase the number of surviving neurons in injured cortex, CA1, and CA3 regions of the ipsilateral hippocampus in the early stage of TBI. These data indicated that FGF2 could assist in preventing neuronal cell death after TBI.

Several years ago, researchers had discovered that FGFs could regulate autophagy in cardiac stem cell differentiation and mouse embryonic fibroblasts [43, 44]. Recently, Wang et al. discovered that FGF2 could regulate autophagy and ubiquitinated protein accumulation in myocardial ischemia mice and spinal cord injury rats [27, 28]. In this study, we further explored the relationship between autophagy and the neuroprotective function of FGF2 in the early stage of TBI. The immunofluorescence study showed that the immunoreactivity of LC3 was downregulated by FGF2, suggesting that FGF2 pretreatment could suppress autophagic cell death. LC3 I and LC3 II are the two forms of LC3, and the ratio of LC3 II to LC3 I is generally used as a biomarker of autophagy [45]. Western blot showed that FGF2 pretreatment can prevent this ratio from increasing. Beclin-1, which played an important role in autophagic cell death, was also downregulated by FGF2. p62 links ubiquitinated protein bodies to LC3 and mediates the autophagic protein degradation, and its accumulation inversely related with autophagic activity [46]. In this study, p62 level was found to be significantly decreased after TBI, while FGF2 could reverse this change. These results suggested that FGF2 could act as an autophagy inhibitor in TBI management.

Autophagy takes an important part in many neurological conditions, including the secondary damage after TBI. Appropriate autophagy can eliminate the aberrant cell components and protein aggregates, so as to maintain cellular homeostasis [14]. In some situations such as subarachnoid hemorrhage, autophagy can play a neuroprotective role and activation of autophagy may protect cortical cells against apoptosis [47]. In TBI, however, whether inducing or inhibiting autophagy can result in neuroprotection is still controversial. On the one hand, the autophagy activator rapamycin or melatonin can protect the brain from TBI-induced damage [13, 48], but on the other hand, the autophagy inhibitor 3-MA or resatorvid could also have similar neuroprotective effects [15, 16]. In the present study, FGF2 could inhibit autophagy and at the same time promoting neurological function, alleviating brain edema, reducing lesion volume, and promoting neuronal survival. This result tallied with previous studies about the autophagy inhibitor 3-MA's neuroprotective effects after TBI [15]. Although the autophagy activator rapamycin may exert a neuroprotective effect after TBI, the results in our experiment showed that coadministration of FGF2 and rapamycin cannot exert a synergistic effect. On the contrary, rapamycin could reverse the benefit effects of FGF2. This result may be related with the dose of rapamycin, and worth further study.

Apoptosis and necrosis are the two major ways of cell death after TBI, and both of them have been linked to autophagy, playing either a prosurvival or prodeath role [49]. Autophagy and apoptosis are the two main types of programmed cell death, and there are closed relations between autophagy and apoptosis. Beclin-1, the essential mediator of autophagy tested in this study, could also be inhibited by antiapoptotic proteins Bcl-2 or Bcl-xl [50, 51]. In addition, autophagy-related gene 5 (Atg5) could increase the susceptibility to apoptotic stimuli [52], and caspase-mediated cleavage of Atg5 and Beclin-1 could switch autophagy into apoptosis [49]. In our study, pretreatment with FGF2 could decrease the number of apoptotic cells and plasma membrane-disrupted cells in injured cortex. These results indicate that FGF2 could alleviate autophagy, apoptosis, and necrosis thus protect cells from various forms of death, at least in rat mild TBI models. Further studies are needed to explore the crosstalk among autophagy, apoptosis, and necrosis after TBI.

In this study, we explored the neuroprotective effects of FGF2 in the early stage of rat mild TBI and discussed the potential mechanisms. Nevertheless, there are several limitations. First, FGF2 is not a specific inhibitor of autophagy, actually it might play neuroprotective roles through various ways such as alleviating blood-brain barrier disruption, as mentioned previously. However, at least, the inhibition of autophagy might take part in FGF2's antiapoptotic and antinecrotic effects. Second, FGF2 was administrated 1 h before TBI, which limited the translational relevance. Whether FGF2 could exert the same effects when delivered after TBI needs further study. Finally, we only investigated the role of FGF2 on autophagy in the first 48 h after TBI, whether FGF2's effect of inhibiting autophagy is associated with its main function of stimulating neural stem/progenitor cell proliferation and differentiation still requires further research.

In summary, the results of this study indicated that FGF2 plays a neuroprotective role in the first 48 h of rat mild TBI through alleviating brain edema and neurological deficits. Pretreatment with FGF2 could also protect cells from various forms of death such as apoptosis or necrosis through inhibition of autophagy. This study extended our understanding of FGF2's neuroprotective effects after TBI. As FGF2 can both attenuate cell death in the early stage after TBI and stimulate neural regeneration and functional recovery later, it may be a promising pharmacological intervention in TBI management.
